# Assessing Epileptogenicity Using Phase-Locked High Frequency Oscillations: A Systematic Comparison of Methods

**DOI:** 10.3389/fneur.2019.01132

**Published:** 2019-10-23

**Authors:** Mojtaba Bandarabadi, Heidemarie Gast, Christian Rummel, Claudio Bassetti, Antoine Adamantidis, Kaspar Schindler, Frederic Zubler

**Affiliations:** ^1^Department of Neurology, Sleep-Wake-Epilepsy Center, Inselspital, University Hospital Bern, University of Bern, Bern, Switzerland; ^2^Department of Neurology, Center for Experimental Neurology, Inselspital, University Hospital Bern, University of Bern, Bern, Switzerland; ^3^Support Center for Advanced Neuroimaging (SCAN), University Institute for Diagnostic and Interventional Neuroradiology, Inselspital, University Hospital Bern, University of Bern, Bern, Switzerland

**Keywords:** epileptogenic zone, presurgical evaluation, electroencephalography, high frequency oscillations, cross-frequency coupling

## Abstract

High frequency oscillations (HFOs) are traditional biomarkers to identify the epileptogenic tissue during presurgical evaluation in pharmacoresistant epileptic patients. Recently, the resection of brain tissue exhibiting coupling between the amplitude of HFOs and the phase of low frequencies demonstrated a more favorable surgical outcome. Here we compare the predictive value of ictal HFOs and four methods for quantifying the ictal phase-amplitude coupling, namely mean vector length, phase-locked high gamma, phase locking value, and modulation index (MI). We analyzed 32 seizures from 16 patients to identify the channels that exhibit HFOs and phase-locked HFOs during seizures. We compared the resection ratio, defined as the percentage of channels exhibiting coupling located in the resected tissue, with the postsurgical outcome. We found that the MI is the only method to show a significant difference between the resection ratios of patients with good and poor outcomes. We further show that the whole seizure, not only the onset, is critical to assess epileptogenicity using the phase-locked HFOs. We postulate that the superiority of MI stems from its capacity to assess coupling of discrete HFO events and its independence from the HFO power. These results confirm that quantitative analysis of HFOs can boost presurgical evaluation and indicate the paramount importance of algorithm selection for clinical applications.

## Introduction

Resection surgery is often the best hope to reach seizure freedom in patients with pharmacoresistant epilepsy (1–3). Ideally, the surgery consists in the resection of brain tissues responsible for seizure generation. Candidates for the epilepsy surgery undergo an extensive multi-modal evaluation using clinical, neuro-radiological and electrophysiological examinations with extracranial and often intracranial EEG recordings (4–9). However, identifying the minimal brain tissue that has to be targeted for surgery, the so-called epileptogenic zone (EZ) (6), remains an unsolved problem.

Several markers have been proved useful in approximating the EZ. One of the most reliable markers for epileptogenic tissue is the presence of sustained low amplitude fast activity (30–120 Hz) at seizure onset (10–14). Fast activity also occurs as brief runs called high frequency oscillations (HFOs > 80 Hz), which can be further separated between ripples (80–250 Hz) and fast ripples (>250 Hz) (15). HFOs were detected in patients and animal models of epilepsy (16), and then linked with epileptogenesis (17), severity of neuron loss (18), and with the location of the seizure onset zone (19).

HFOs became a potent marker of the EZ after several studies reported correlation between favorable postsurgical outcome and the resection of brain tissue generating interictal, perictal, or ictal HFOs (12, 20–31), but controversies were also raised (31). Several recent studies reported that not only the presence of HFOs, but also the timing of HFO occurrence with respect to slower oscillations, e.g., epileptiform activity and sleep slow waves, is important for EZ identification (32–38).

Different methods exist to quantify the dependency between the phase of slow oscillations and the amplitude of HFOs, referred to as phase-amplitude cross-frequency coupling. Four of them have provided predictive value for postsurgical outcome when applied to intracranial EEG and. These four measures include the mean vector length, also used with different terms (39, 40), phase locking value (37), phase-locked high gamma (33, 34), and modulation index (36, 38, 41). However, a systematic comparison between these methods is still missing. Here we assess these four different phase-amplitude coupling methods, as well as the amplitude of HFOs (regardless of coupling), for predicting the outcome after epilepsy surgery.

## Methods

### Participants

Sixteen adult patients with pharmacoresistant epilepsy (11 females; median age 31 y, range 19–59 y) were included in this study. This dataset has been previously described in detail (8, 42). The intracranial EEG recordings were performed during presurgical evaluation at the University Hospital of Bern (Inselspital). All patients then underwent resective epilepsy surgery after presurgical evaluation. Patients were monitored for several years after surgery and outcome of surgery was classified according to the Engel Epilepsy Surgery Outcome Scale. All participants signed written informed consent. This study was approved by the Ethics committee of the Canton of Bern (license number 2017-00697).

### Data Acquisition and Preprocessing

Intracranial recordings were performed with strip, grid or depth electrodes (Ad-Tech Medical, Racine, WI) with an extracranial reference electrode placed over the frontocentral region (between Fz and Cz). The distance between neighboring contacts was 1 cm. The sampling rate of data was either 512 or 1,024 Hz, depending on the number of channels. In the latter case, data were downsampled to 512 Hz after applying the Chebyshev Type I low-pass filter (“decimate” function, MATLAB, MathWorks Natick, MA). Traces were visually inspected by experienced epileptologists (HG, KS, FZ) and channels with permanent artifacts were excluded. We re-referenced all intracranial EEG recordings against the median of all channels free of permanent artifacts.

### Assessment of Phase-Locked HFOs

**Figure 1:**
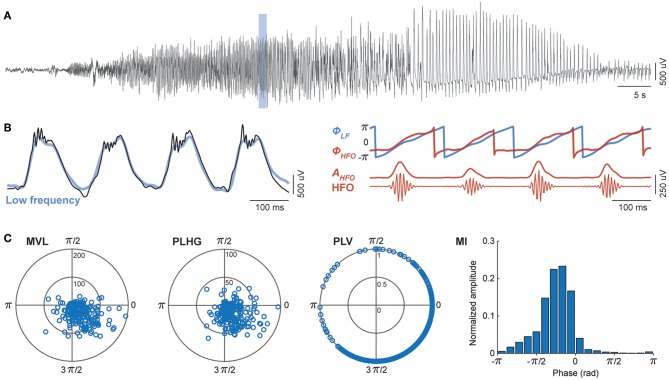
Assessment of phase-locked HFOs using four different methods. **(A)** Representative channel of intracranial EEG during an epileptic seizure. **(B)** A temporal magnification showing high frequency oscillations (HFO) superimposed onto rhythmic low frequency (LF) signal (black). All four methods involve band-pass filtering in slow (blue) and fast (red) frequency bands. The phase-amplitude cross-frequency coupling measures are estimated from the phase of low frequency components (Φ_*LF*_), and either the phase (Φ_*HFO*_) or the amplitude (*A*_*HFO*_) of HFO envelope. **(C)** The mean vector length (MVL), phase-locked high gamma (PLHG), phase-locking value (PLV), and modulation index (MI) differ in the way they quantify the non-uniformity of HFO power during LF cycles (see section Assessment of Phase-Locked HFOs of the main text).

We evaluated the performance of four different measures of cross-frequency coupling between low frequency and HFOs, namely mean vector length (43), phase-locking value (44), phase-locked high gamma (33), and modulation index (45). All four methods have in common the band-passed filtering of the intracranial EEG signals, once in low frequency, and once in the HFO range. The methods differ in the way a potential relationship between the phase of the low frequency band and activity in HFO band is quantified (Figure 1).

We filtered intracranial EEG signals between 4 and 30 Hz for the low frequency components and 80–150 Hz for the HFO band. We designed finite impulse response (FIR) bandpass filters using the window-based approach (“fir1” function, MATLAB), with an order equal to three cycles of the low cutoff frequency. To eliminate phase distortions, we filtered EEG signals in the both forward and reverse directions (“filtfilt” function, MATLAB). We used wideband filtering for low frequency components to preserve the shape of epileptic spike-waves as previously suggested (33). We then calculated the instantaneous phase of the low frequency (Φ_*LF*_), the instantaneous envelope of the HFOs (*A*_*HFO*_), and the instantaneous phase of HFOs envelope (Φ_*HFO*_) using the Hilbert transform. For comparison, we also computed the HFOs power, independently of its relation to the phase of the slow frequency, as the average of the HFOs envelope in each segment of recording (Supplementary File).

#### Mean Vector Length

The first coupling measure, mean vector length (MVL), uses the phase of low frequency and the amplitude of the envelope of the HFO band. At each time step, a complex number is defined, of which the module is the instantaneous envelope of the HFO (*A*_*HFO*_), and the phase is the instantaneous phase of the low frequency band (Φ_*LF*_). The MVL is the absolute value of the average of all complex numbers obtained during a given time window,

(1) $$\begin{array}{l} MVL=\;\left|1/n\;*\sum_{1}^{n}A_{HFO}\;*\;e^{\Phi_{LF}}\right| \end{array}$$

where *n* is the number of samples within the window.

#### Phase-Locked High Gamma

The second measure, phase-locked high gamma (PLHG), uses the phase of the low frequency, the HFO envelope, and the phase of the HFO envelope (33). The instantaneous phase difference between the low frequency and the HFO envelope is first calculated and then projected onto a unit circle. The instantaneous phase difference is then multiplied by the instantaneous HFO envelope, which is normalized by dividing to mean of HFO envelope in a reference segment (30 s preictal baseline). The PLHG measure is the absolute value of the average of these complex values within a segment and calculated as,

(2) $$\begin{array}{l} PLHG=\;\left|1/n\;*\sum_{1}^{n}{A_{NormHFO}\;*\;e}^{\Phi_{LF}-\Phi_{HFO}}\right| \end{array}$$

#### Phase-Locking Value

The third method, phase-locking value (PLV), uses the phase of the low frequency and the phase of the HFO envelope. PLV is obtained by averaging the instantaneous phase differences between the low frequency band and the HFO envelope signal, which are projected onto a unit circle in the complex plane and calculated as,

(3) $$\begin{array}{l} PLV=\;\left|1/n\;*\sum_{1}^{n}e^{\Phi_{LF}-\Phi_{HFO}}\right| \end{array}$$

In contrast to the other measures, PLV is always in the [0–1] interval, where one indicates completely phase-locked signals and zero the absence of phase locking.

#### Modulation Index

The last method, modulation index (MI), uses the phase of the low frequency and the HFO envelope. To calculate MI, the phase of the low frequency is discretized into *N* bins of equal sizes. We used *N* = 18, each bin consisting of 20°, as suggested in the original papers (45, 46). The average amplitude of the HFO envelope is then calculated inside each bin, where for bin *i* we averaged the HFO envelope over all periods during which the phase of the low frequency signal is between *2*π*(i*−*1)/N* and *2*π^*^i/N. The resulting phase-amplitude histogram (*P*) is then compared with the uniform distribution (*U*) using the Kullback-Leibler divergence,

(4) $$\begin{array}{l} D_{KL}\left(P,U\right)=\;\sum_{i=1}^{N}P\left(i\right)\;*\;log\left[P\left(i\right)/U\left(i\right)\right] \end{array}$$

which in turn is normalized by log(*N*) to obtain the modulation index,

(5) $$\begin{array}{l} MI=\;D_{KL}/\log\left(N\right) \end{array}$$

To consider non-uniform distribution of low frequency phase, we corrected the calculated phase-amplitude distribution by subtracting it from phase distribution.

### Evaluation of the Measures

To assess the phase-locking dynamics during epileptic seizures, we calculated measures using a 3-s moving window with 333 ms moving step, as in Weiss et al. (33), and then smoothed the extracted measures using a 10-point moving window. We applied bandpass filtering before segmentation to eliminate edge effects. For each measure, we quantified the percentage of channels passing a certain threshold during the course of a seizure that recorded from the resected area (the resection ratio). We defined the threshold for each seizure/measure individually as 2.5 standard deviations above the mean (2.5 SD + mean) obtained from all channels and ictal time segments of that seizure. We then compared the resection ratio between seizures of patients with good (Engel Class I-II) and poor (Engel IV) outcomes. No patient had an Engel Class III outcome. Furthermore, we followed the method proposed by Weiss et al. (34), where they only considered early channels that reached a given threshold to calculate the resection ratio. The authors selected the first four channels reaching the threshold, as it was the average number of resected channels in their population study. We performed this analysis using the first nine channels, which is the average number of resected channels in our group of patients. We analyzed two seizures per subject (the minimum number of seizures recorded per patient) in order to avoid bias toward patients with many seizures.

### Statistical Analysis

We compared the statistical difference between patients with good and poor outcomes with the Mann-Whitney U-test, using the Statistics and Machine Learning Toolbox from MATLAB. We adjusted the statistical significance *p*-value from 0.05 to 0.01 to correct for multiple comparisons (Bonferroni correction for five tests).

## Results

**Figure 2:**
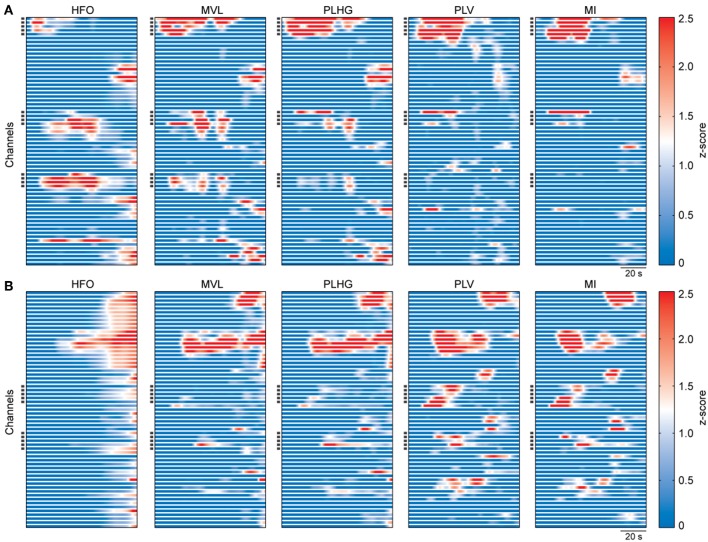
Representative examples of estimated measures during seizures. **(A)** Calculated measures from intracranial EEG recordings of a patient with outcome Engel I. The graphs show the whole seizure, from onset to termination. The measures were obtained using a moving window of 3 s with 333 ms moving step, and then smoothed using a 20-sample moving window. Values of each graph were z-scored using all channels and ictal time segments of the representative seizure. Each horizontal line represents one channel. Dark squares on the y-axis indicate the resected channels during surgery. **(B)** Same as **(A)**, but for a patient with outcome Engel IV.

We analyzed intracranial EEG recordings of 32 epileptic seizures from 16 patients. We calculated the HFO power and the four above-mentioned measures to assess the phase locking of HFOs to the low frequency components of the signal. Figure 2 shows examples of these measures obtained from seizures of two patients with Engel I and IV outcomes.

### Comparison Between Measures During the Whole Seizure

**Figure 3:**
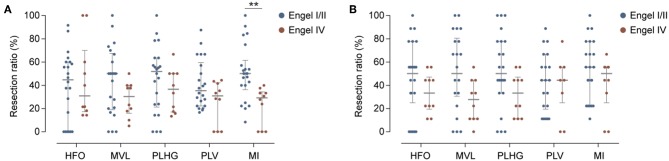
Evaluation of phase-locked HFO measures. **(A)** Percentage of channels reaching the threshold for HFOs power or phase-locked HFO levels that were resected in patients with good (blue) or poor (red) outcome. Horizontal and vertical bars indicate the median and interquartile range, respectively. Only the MI measure showed significant differences between two groups. Detailed results [median (inter-quartile range)]: Engel I/II vs. Engel IV: HFOs: 44.8% (0–60) vs. 30.9% (18–60), *P* = 0.8; MVL: 50% (20–66) vs. 30.3% (17–40), *P* = 0.13; PLHG: 51.9% (23–63) vs. 36.6% (17–50), *P* = 0.28; PLV: 35.4% (22–57) vs. 30.1% (0–41), *P* = 0.22; MI: 50% (40–57) vs. 29.3% (0–33); *P* = 0.0024**). **(B)** Same as **(A)**, but for early nine channels. None of the measures showed significant differences between two groups. Detailed results: Engel I/II vs. Engel IV: HFOs: 50% (33–77) vs. 33.3% (22–44), *P* = 0.26; MVL: 50% (33–77) vs. 27.7% (11–44), *P* = 0.052; PLV: 44.4% (22–66) vs. 44.4% (33–55), *P* = 0.61; PLHG: 50% (33–77) vs. 33.3% (11–44), *P* = 0.08; MI: 55% (22–77) vs. 50% (IQR: 33–55), *P* = 0.27.

For each measure, we calculated the resection ratio, which is the percentage of channels reaching the threshold at any time during the seizure that were resected. We then compared the resection ratio between patients with good and poor outcome. Figure 3A shows the results of this analysis. The difference was statistically significant only for the MI, for which a median of 50%, with 40–57% interquartile range (IQR), of channels were resected in patients with good outcome, while for patients with poor outcome a median of 29.3% (IQR: 0–33%) of channels were resected (*P* = 0.0024). The trend was similar for the other coupling measures as well as for HFO power, however without reaching statistical significance.

### Comparison Between Measures Using Early Channels

When considering only the first nine channels to reach the threshold level during seizures (“early channels”), we found that the resection ratio did not significantly differ between patients with good and poor outcome (Figure 3B). The results were close to the uncorrected significance level for MVL, with a percentage of channel removal of 50% (IQR: 33.3–77.8) in case of good outcome vs. 27.7% (IQR: 11.1–44.4) in case of poor outcome (*P* = 0.052).

### Correlation Between Phase-Locked HFO Measures and HFO Power

**Figure 4:**
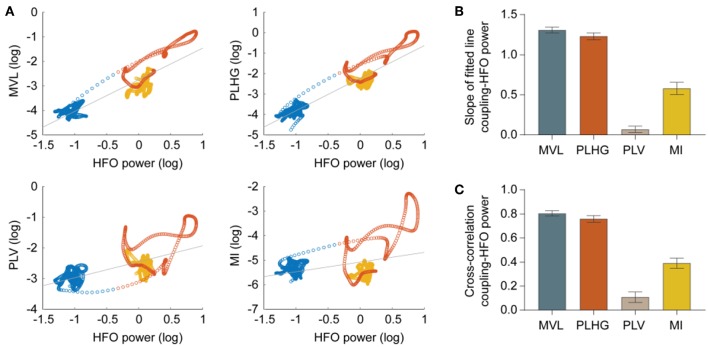
Correlation between phase-locked HFO measures and HFO power. **(A)** Scatter plots show relations between measures and HFO power during preictal (blue circles), ictal (red), and postictal (yellow) periods for a representative seizure and channel. Each circle represents values obtained from a 3-s moving window with 333 ms moving step. The gray line is the fitted to the samples. **(B)** Quantification of slope of the fitted lines in the scatter plots, estimated from all channels of 32 seizures (MVL = 1.31 ± 0.03; PLHG = 1.23 ± 0.04; PLV = 0.07 ± 0.04; MI = 0.58 ± 0.07; *n* = 2,034 channels). **(C)** Normalized cross-correlation between the coupling measures and HFO power (MVL = 0.80 ± 0.02; PLHG = 0.76 ± 0.03; PLV = 0.11 ± 0.04; MI = 0.39 ± 0.04; *n* = 2,034 channels). Both the slope and cross-correlation analyses indicate that the MVL and PLHG measures are highly biased toward HFO power. Error bars indicate mean ± standard error mean.

We assessed the dependency of each coupling measure to the HFO power using two approaches: First with the slope of the fitted lines between each coupling measure and HFO power in a logarithmic scale (Figures 4A,B), second, with the cross-correlation (Pearson correlation) between time series of the coupling measures and HFO power (Figure 4C). Both the slope and cross-correlation analyses indicate that the MVL and PLHG measures are highly influenced by the HFO power.

## Discussion

Recent studies reported that phase-locked HFOs during ictal EEG contribute to identification of epileptogenic brain tissues more accurately than HFOs alone. Here we evaluated and compared four different phase-amplitude coupling measures, as well as the HFOs power, for predicting the outcome of epilepsy surgery in terms of seizure control. For each measure, we compared the resection ratio, namely the proportion of channels showing high coupling values that were within the resected area, in patients with good vs. poor outcome. MI was the only measure showing a significant difference between the two groups. We also found that the whole seizure, not only the onset, should be considered when selecting channels with high coupling value.

### Specificity of the Modulation Index

Ictal HFOs are discrete events. Therefore, the phase of HFO envelope is meaningful only during the HFOs and is ill defined in the absence of HFOs. Moreover, ictal HFOs occur predominantly in specific phases of spike waves (Figure 1C). However, their duration is usually shorter than the duration of a spike wave, so that comparing the instantaneous phase of HFOs and spike waves is misleading, as they have different cycle lengths. These two points make the PLV and PLHG measures, which rely on the instantaneous phase of the HFO envelope, ill-defined in the context of ictal HFOs. Moreover, the MVL and PLHG measures use the amplitude of the HFO envelope, thus are highly biased toward HFO power (Figure 4). This bares the risk that a channel with high HFO power, but low coupling levels, shows a higher coupling value according to these measures than a channel with low HFO power, but high coupling levels. Together, these mechanisms contribute to the superiority of MI compared to the other measures for identifying epileptogenic brain regions. Indeed, the MI is the only measure that satisfies two crucial requirements for the current problem; (1) it can capture coupling patterns of discrete events by summing up the HFO envelope at the specific phase of low frequency components, (2) it uses the normalized phase-amplitude histograms and therefore is independent of HFO power.

### Definition of Low Frequency Components

We considered a relatively wide band (4–30 Hz) as low frequency band. The reason was to preserve the characteristic shape of EEG signals during seizures (33), where sharp waves coexist with low frequency waves. This is especially important considering the fact that ictal epileptic signals have asymmetric shapes (47). Filtering in more narrow frequency bands would constitute an important issue for accurate estimation of their phase. However, we also examined several other frequency bands (0.5–4, 1–16, and 1–30 Hz). The separation was not superior to the original choice of 4–30 Hz. Of note, the MI remained the best, or was very close to the best predictor also for these different frequency bands.

### Whole Seizure vs. Early Channels

Considering coupling at any time during the course of the seizure yielded better separation than restricting the analysis to the first channels reaching the threshold level for coupling. This is in contrast with previous findings by Weiss et al. (33). A probable explanation is that channels in the seizure onset zone often register low amplitude fast oscillations at seizure onset. Due to the frequently observed absence of low frequency activity during seizure onset in these channels, they cannot exhibit any cross-frequency coupling. Expanding the window to the whole seizure offers the possibility for channels in the EZ to reach the coupling threshold at a later point, when periodic or rhythmic low frequency epileptiform discharges appear (Figure 1A). From the clinical perspective, considering coupling at any point during a seizure also increases the chance to obtain additional information to the merely visual analysis for delineating the resection area, which currently mainly focuses on seizure onset.

### Importance of the Algorithm Selection

Despite overwhelming evidence that quantitative EEG analysis, in particular HFOs analysis, improves presurgical evaluation of epilepsy, these methods have not been widely integrated into clinical setting (48). The fact that various groups use different preprocessing methods, quantification algorithms, and outcome evaluation, rends replication of findings difficult (30). Our results confirm that the choice of algorithm is of utmost relevance. Previous work suggested that outcome-oriented studies using HFOs should report the details of HFO detection algorithm to allow for comparisons (30). We further suggest that studies should indicate reasons for choosing one particular algorithm and discuss the limitations and specificities of the algorithm compared to other existing methods. This step would lead to a better characterization of the various methods, which is a prerequisite for an application in clinical setting.

### Strengths and Limitations

To the best of our knowledge, this is the first study to compare systematically different methods for assessing coupling between low frequencies and HFOs on the same epilepsy patients. Data come from a very well-characterized collective of patients who have been monitored for several years after resection surgery and documented in previous studies. We discussed the advantages and drawbacks of different methods to inform neurophysiologists about choosing the proper method for the specific problem they address. One limitation of our study is that we did not investigate HFOs at frequencies higher than 150 Hz because the original sampling rate of the EEG recordings hindered this analysis. Another limitation is the relatively low number of patients (16); however, the number of seizures did not prevent us to reach a very high level of statistical significance.

## Conclusion

Presurgical evaluation remains a very challenging task. Clinical workup is slowly incorporating the quantitative approaches. This study provided experimental and theoretical arguments in favor of using the MI when quantifying the low frequency-HFO coupling during ictal events for identifying epileptogenic tissue. However, it is of paramount importance to be aware of the limitations of a single quantitative method. We provided the MATLAB scripts of the investigated methods (Supplementary File), so that epileptologists can apply them on other datasets to better characterize their role for presurgical evaluation.

## Data Availability Statement

The dataset for this paper is publicly available online at http://ieeg-swez.ethz.ch/. The scripts used to perform the analysis can be found as Supplementary Data.

## Ethics Statement

All participants signed written informed consent. This study was approved by the Ethics committee of the Canton of Bern.

## Author Contributions

MB, AA, KS, and FZ contributed in the conception and design of the study. HG, CR, KS, and FZ participated in acquiring, annotating, and preprocessing of data. MB and FZ performed the analysis. MB, KS, and FZ drafted the manuscript. All authors critically revised the manuscript and made a substantial contribution to interpreting data, and approved the manuscript.

### Conflict of Interest

The authors declare that the research was conducted in the absence of any commercial or financial relationships that could be construed as a potential conflict of interest.
